# Application of iron-loaded activated carbon electrodes for electrokinetic remediation of chromium-contaminated soil in a three-dimensional electrode system

**DOI:** 10.1038/s41598-018-24138-z

**Published:** 2018-04-10

**Authors:** Yujie Yan, Fengjiao Xue, Faheem Muhammad, Lin Yu, Feng Xu, Binquan Jiao, YanChyuan Shiau, Dongwei Li

**Affiliations:** 1 State Key Laboratory of Coal Mine Disaster Dynamics and Control, Chongqing University, Chongqing, 400044 China; 2 City College of Science and Technology, Chongqing University, Chongqing, 400044 China; 3 Dept. of Construction Management, Chung Hua University, No. 707, Wufu Rd., Sec. 2, Hsinchu, 30012 Taiwan; 4 Chongqing Solid Waste Management Center, Chongqing, 400044 China

## Abstract

Hexavalent chromium from industrial residues is highly mobile in soil and can lead to the contamination of groundwater through runoff and leaching after rainfall. This paper focuses on the three-dimensional (3D) electrokinetic remediation (EKR) of chromium-contaminated soil from an industrial site. Activated carbon particles coupled with Fe ions (AC-Fe) were used as the third electrode. The optimum dose ratio of the electrode particles and remediation time were selected on the basis of single-factor experiments. X-ray photoelectron spectroscopy (XPS) analysis was carried out to explore the reduction of Cr(VI) on the surface of the electrode particles (AC-Fe). The results showed that AC-Fe had a positive effect on Cr(VI) reduction with a removal rate of 80.2%, which was achieved after 10 d by using a 5% dose of electrode particles. Finally, it was concluded that the removal mechanism combined the processes of electromigration, electrosorption/adsorption and reduction of Cr(VI) in the 3D EKR system.

## Introduction

Chromium is widely used in a variety of industrial process. As a result, a large amount of chromium-contaminated waste is produced, which pollute the soil, water and atmosphere^1–4^. Chromium-contaminated soil has become a leading concern in China and across the world^5–8^. Electrokinetic remediation (EKR) has become a promising technique for removing chromium from contaminated soils. This technique is based on an electrolytic cell, in which both electrodes are supplied with a low-voltage direct current or a low potential gradient to treat contaminated soil^9–12^. A number of chemical and physical reactions occur in the electrolytic cell, including electrolysis, desorption, dissolution, electromigration, electroosmosis and electrophoresis, which in turn affect the soil physicochemical characteristics, such as the pH, electrical conductivity and zeta potential^13^. However, EKR has limitations when treating soils with a low electrical conductivity, poor permeability and heterogeneous subsurface^14,15^.

In recent years, considerable attention has been paid to three-dimensional (3D) EKR. The three-dimensional cell is developed from a traditional two-dimensional (2D) cell by filling it with particulate material. The third particle-based electrode has a large surface area, which enhances the mass transfer rate and adsorption capability. Therefore, the 3D cell has a higher current efficiency and removal rate than the traditional 2D cell^16–19^. The three-dimensional EKR technique has been widely used to remove organic and inorganic substances from industrial wastewater^18,20–23^, indicating its practical implication^19^. However, few studies have been reported on 3D EKR for treating solid waste, such as contaminated soils.

In this study, 3D EKR experiments were carried out to remediate chromium-contaminated soil from an abandoned industrial site by using iron-loaded activated carbon (AC-Fe) as the third electrode. Two single-factor experiments were conducted to explore the effects of the particle electrode dose and treatment time on the Cr(VI) and total Cr removal efficiencies. In addition, AC and AC-Fe were subjected to Brunauer-Emmett-Teller (BET) surface area analysis and scanning electron microscope (SEM) analysis to examine the specific surface area and microstructural changes, respectively. X-ray photoelectron spectroscopy (XPS) was performed on the particle-based electrode and the soil to explore the Cr removal mechanism.

## Materials and Methods

### Materials and chemicals

Activated carbon (AC) synthesized from coconut shell was purchased from Chengde, Hebei, China. This AC was sieved through a 6 mm standard sieve, washed with deionized water, and then dried. The chromium-contaminated soil sample was collected from an abandoned industrial site located in Chongqing, China, dried, sieved through 100-mesh sieves, and then stored in sealed bags at room temperature. The elemental composition of the soil was determined by X-ray fluorescence (XRF), as shown in Table 1. In addition, the physicochemical characteristics of the soil samples are shown in Table 2. All the chemicals used were of analytical grade, and the water used in the experiment was deionized.

**Table 1:** Elemental composition of the original chromium-contaminated soil.

Ca	O	Si	Al	Cr	Fe	Mg	S	K
12.2	42.9	11.9	7.3	9.4	7.6	5.8	1.4	0.6
**Na**	**Ti**	**Ni**	**V**	**Sr**	**P**	**Zn**	**Co**	**Zr**
0.3	0.3	0.06	0.06	0.05	0.04	0.04	0.04	0.01

**Table 2:** Physical and chemical characteristics of the soil sample.

Property	Value	Method
pH	8.07	1:5 Soil/water slurry
Conductivity	51.1 (mS/cm)	1:5 Soil/water slurry
Cr(VI) concentration in soil	1172.8 (mg/kg)	Alkaline digestion
Total Cr concentration	14133.0 (mg/kg)	Acid digestion

### Preparation of AC-Fe electrode particles

The AC-Fe electrode particles were prepared using an impregnation method^24–26^. The dried AC was immersed in a ferrous sulfate heptahydrate (FeSO_4_•7H_2_O) solution with a mass fraction of 10% for 10 h. The solids obtained after filtration were dried at 90 °C in the presence of N_2._ Subsequently, the dried solids were calcined in a tube furnace at 350 °C for 2 h in a N_2_ atmosphere. The synthesized electrode particles were cooled and stored in a sealed plastic bag for further use. In addition, the morphologies of the AC and AC-Fe electrode particles were analysed by SEM (TESCAN MIRA 3 FE-SEM, America). The changes in the surfaces of the synthesized materials prior to and after the experiments were analysed.

### Aqueous equilibrium adsorption tests

Aqueous equilibrium adsorption tests were conducted to evaluate the adsorption and reduction capacity of AC and AC-Fe. For this purpose, a K_2_Cr_2_O_7_ solution was prepared by dissolving 39.4 mg of K_2_Cr_2_O_7_ in 1000 mL of deionized water. This K_2_Cr_2_O_7_ solution was used to prepare a solution containing 13.94 mg/L of Cr(VI), which was equivalent to the Cr(VI) leaching concentration of the soil. In addition, CaCl_2_ of 41.6 mg/L, AlCl_3_ of 59.3 mg/L and MgCl_2_ of 39.58 mg/L were added into the K_2_Cr_2_O_7_ solution according to the contents of Ca^2+^, Mg^2+^ and Al^3+^ in the soil sample^27^.

0.1 g of each dried AC and AC-Fe were added into 30 mL of the K_2_Cr_2_O_7_ solution to achieve the adsorption equilibrium. The pH of the solution was adjusted in the range of 3.0–11.0 to evaluate its effect on the equilibrium adsorption and reduction of AC-Fe. All the tests were carried out in conical flasks that were placed on an oscillator operated at a rate of 120 rpm at 25 °C. After 24 h, the mixtures were filtered through 0.45 μm filter membranes. The concentration of Cr(VI) in the filtrate was determined by a UV spectrophotometer, and the removal rate is calculated by the following formula (1):

1 $${\rm{C}}=\frac{{{\rm{C}}}_{0}-{{\rm{C}}}_{{\rm{a}}}}{{{\rm{C}}}_{0}}\times {\rm{100}}$$

where C_0_ and C_a_ (mg/L) are concentrations of Cr(VI) in initial solution (without AC and AC-Fe) and equilibrium solution (with AC and AC-Fe).

### Electrokinetic remediation experiments

**Figure 1:**
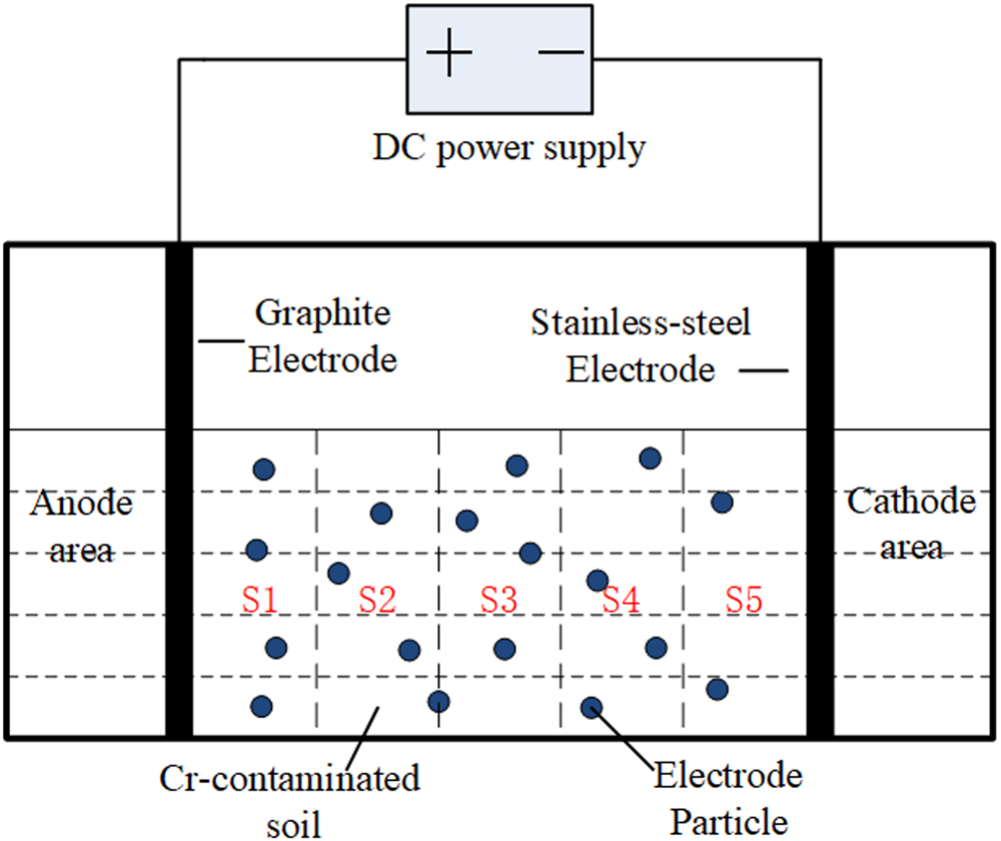
Schematic diagram of the EKR experimental device.

The device used in the EKR experiments is illustrated in Fig. 1. This device consists of two main parts: the soil treatment chamber and the electrode compartment. The soil treatment chamber or sample region (100 mm × 60 mm × 80 mm) was evenly divided into 5 regions (S1, S2, S3, S4 and S5) from the anode to cathode. Graphite and stainless-steel plates were used as the anode and cathode, respectively, while the synthetic AC-Fe particles were used as the third electrode. The removal efficiency of Cr(VI) was investigated in conventional 2D EKR (with a dose of 0) and 3D EKR experiments. Several dose ratios of AC-Fe (1%, 3%, 5%, 7%, 9% and 11%) were used in the 3D electrokinetic remediation experiments to assess the effect of the dose on the removal efficiency of Cr(VI). Except for dose ratios in the 3D EKR experiments, all the other experimental conditions remained the same (Table 3).

**Table 3:** Single-factor experimental conditions regarding the dose ratio.

Test number		—	T1	T2	T3	T4	T5	T6
Dose ratio of AC-Fe (%)		0	1	3	5	7	9	11
Electrode	Anode	Graphite plate (10 mm * 60 mm * 80 mm)						
	Cathode	Stainless-steel plate (2 mm * 60 mm * 80 mm)						
Experimental conditions	Voltage gradient (V/cm)	1						
	Proposing day (d)	5						
Electrolyte		Na_2_SO_4_ (0.1 mol/L)						

After determining the optimum dose of AC-Fe (5%), another single-factor experiment was carried out to optimize the remediation time. In this single-factor experiment, the time was chosen as the variable factor (7 d, 10 d and 13 d), while other factors like the voltage gradient and dosing ratio were held constant.

### Analytical method

The soil electrical conductivity (EC) and pH were measured by an EC metre (DDSJ-308A, Leici, China) and a pH metre (PHS-3C, Leici, China), respectively. The changes in the elemental forms and chemical oxidation states of the particle electrode before and after the experiment were examined by XPS (ESCALAB 250Xi, America). The specific surface area of the activated carbon was determined by the BET method (Quadrasorb 2MP, America).

The concentrations of Cr(VI) and total Cr in the soil were measured by an ultraviolet spectrophotometer (UV-2100, Japan) and atomic absorption spectrometer (AAS, AA-6300C, Japan), respectively.

## Results and Discussion

### Characterization of materials

**Figure 2:**
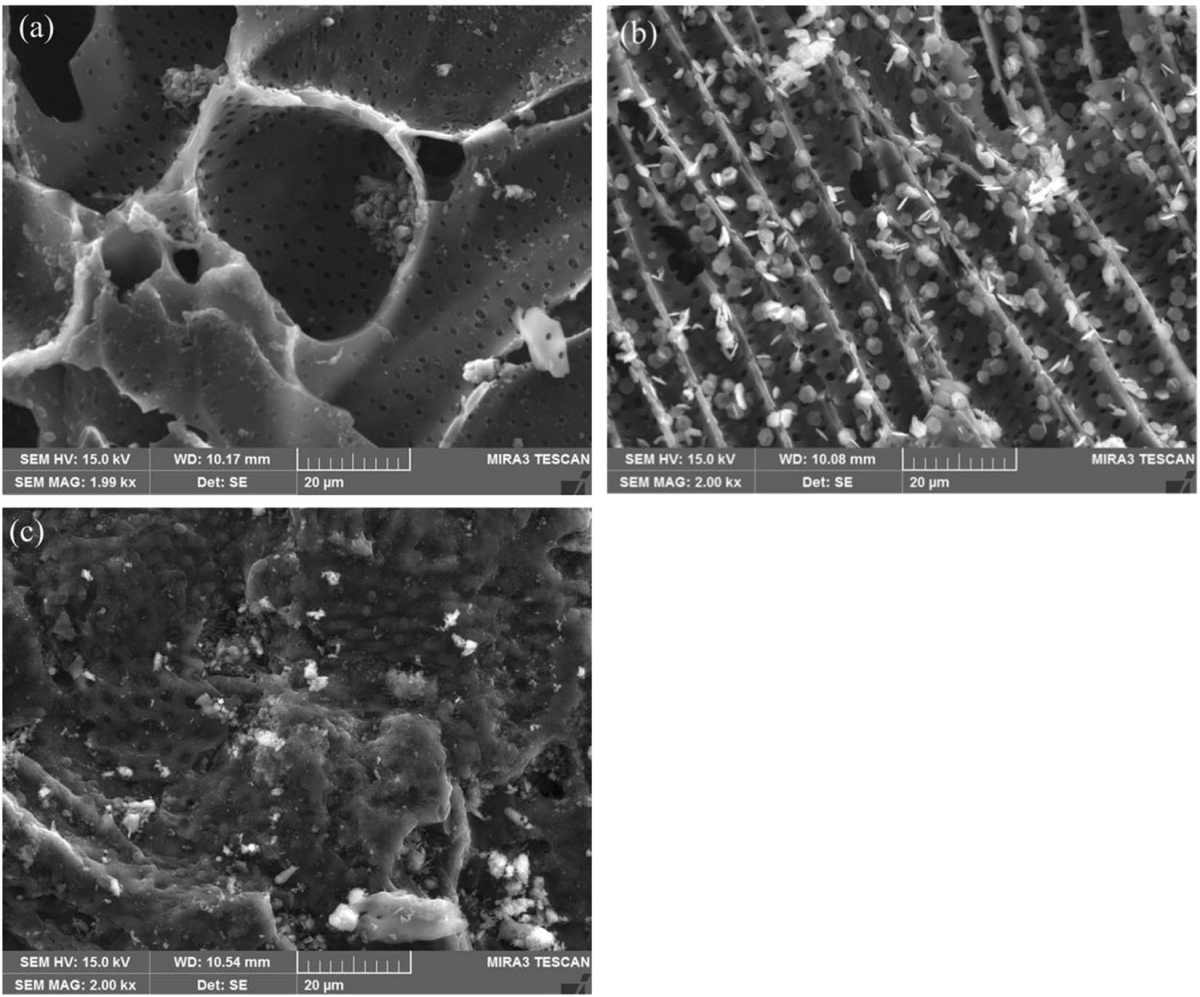
SEM images of (**a**) AC, (**b**) AC-Fe before EKR and (**c**) AC-Fe after EKR.

The morphologies of AC and AC-Fe before and after the experiments are shown in Fig. 2. As seen in Fig. 2(a), the unloaded AC had a developed pore structure, which indicated its capability of adsorption. Figure 2(b) indicated that iron particles were successfully loaded on the surface of the activated carbon. In addition, further analysis of the particles was performed by XPS. Figure 2(c) shows the morphology of the AC-Fe particles recycled after the EKR experiments, in which the pore structures could not be observed due to the substantial amount of contaminants adsorbed on the surface of the AC-Fe particles. The BET specific surface areas of AC and AC-Fe are 847.86 m^2^/g and 728.43 m^2^/g, respectively.

### Aqueous equilibrium adsorption tests

**Figure 3:**
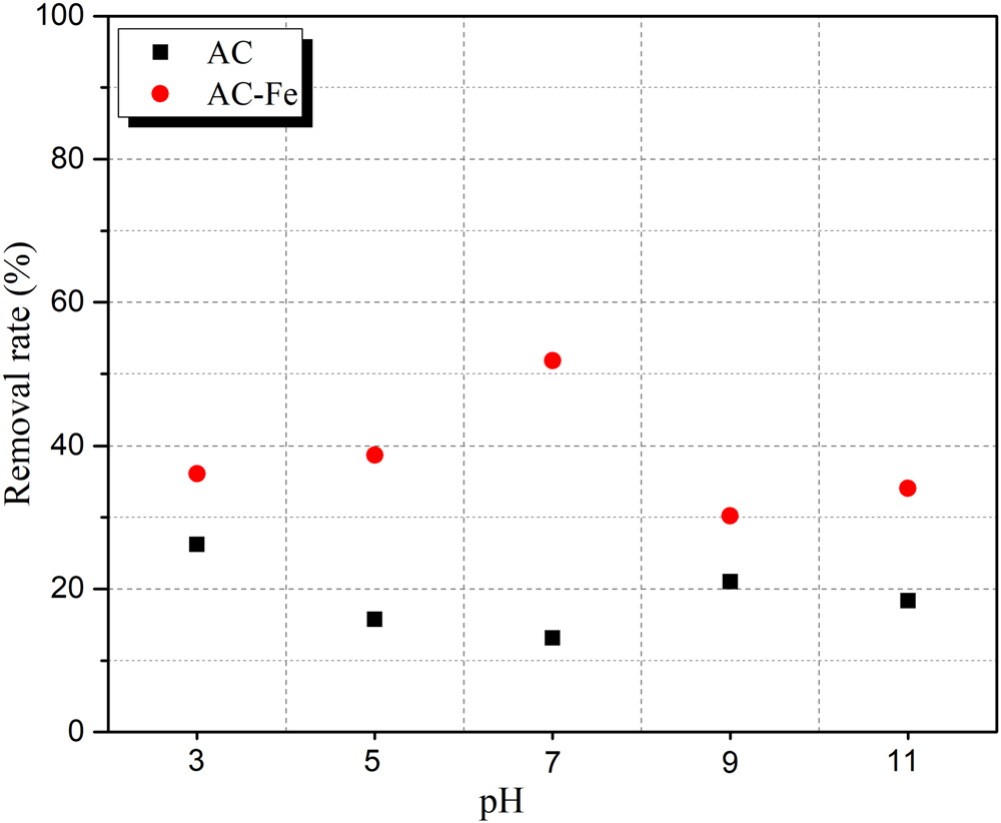
Cr removal rate at different pH levels.

Batch tests of the aqueous equilibrium adsorption were conducted to explore the effects of the synthetic material (AC-Fe) on Cr(VI) reduction at different pH values. The removal rate of Cr(VI) in the aqueous phase are shown in Fig. 3. The Cr(VI) removal efficiency of AC was mainly attributed to adsorption, and thus, AC exhibited a low removal rate. For AC-Fe, the removal rate of Cr(VI) was significantly larger than that of AC adsorption, which indicated that Cr(VI) as reduced by Fe^2+^. For the pH effect, less Cr reduction was observed at higher pH values. This result might be due to the competition of OH^−^ with Cr(VI), in which more OH^−^ adsorbed and reacted on the AC-Fe sites; hence a lower removal rate of Cr(VI) was observed in the alkaline environment^27–30^. In addition, the coprecipitation of Al^3+^, Ca^2+^ and Mg^2+^ in the alkaline environment was also a limiting factor for the reduction of Cr(VI).

### Effect of the particle dose ratio on removal efficiency

#### Two-dimensional EKR experiments

**Figure 4:**
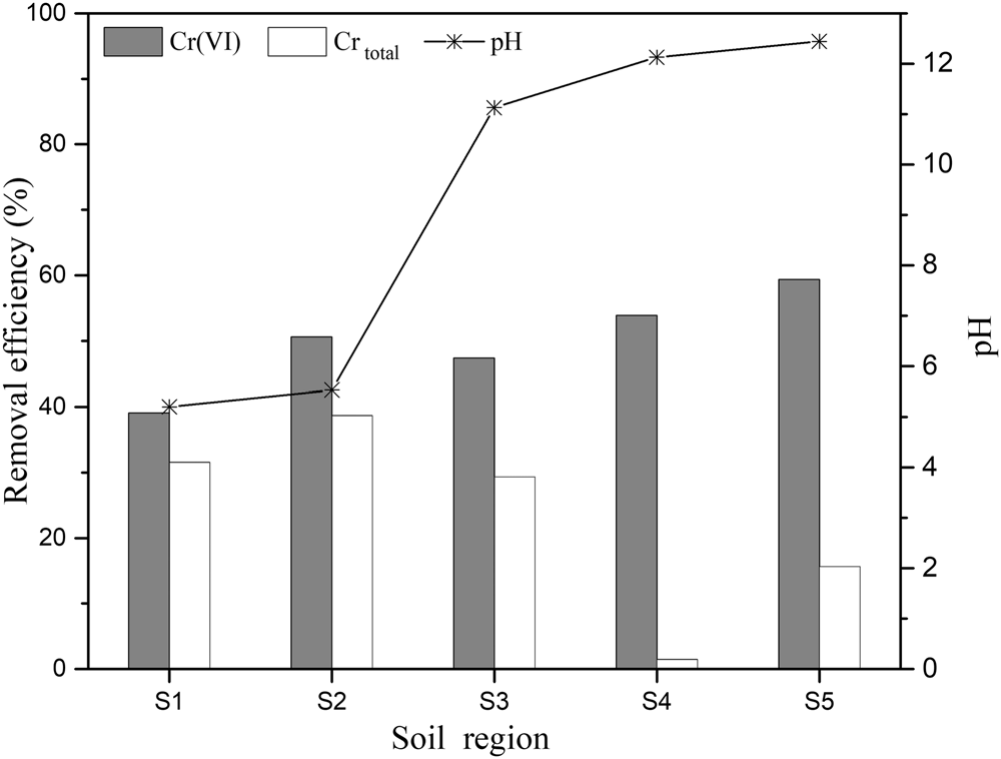
Removal efficiency and pH in the 2D EKR experiment.

For the 2D EKR test (with a dose ratio of 0), the removal efficiencies and pH values of the five sample regions after 5 d are shown in Fig. 4. The lowest Cr(VI) removal efficiency was observed in the sample region closest to the anode, and an increasing trend was observed for the sample regions moving towards the cathode (Fig. 4). This trend occurred because during the electrical remediation process, hexavalent chromium mainly existed in the form of oxyanions, such as CrO_4_^2−^, Cr_2_O^7−^, HCrO_4_^−^, and H_2_CrO_4_^−^. These predominant species of Cr(VI) migrated in the direction of the anode under the force of the applied electric field, resulting in the accumulation of Cr(VI) in the soil region closest to the anode. It was concluded that during the process of electrical remediation, Cr(VI) removal occurred through (i) the reduction of Cr(VI) to Cr(III) and (ii) the existence of Cr(VI) as oxyanions and the migration of these anions towards the anode. The latter was also the reason for the trend in the removal of the total Cr. In the conventional 2D EKR experiments, the maximum Cr(VI) removal rate of 59.4% was obtained in region S5, and the minimum removal ratio of 39.0% was obtained in S1, which indicated that Cr(VI) accumulated in S1 near the anode; this phenomenon was similar to the results reported previously^13,14^. In the case of the total Cr, the opposite trend was observed: the highest total Cr removal rate of 38.6% was obtained in S2, and the lowest ratio of 1.5% was obtained in 4. As shown in Fig. 4, regions S4 and S5 had higher removal rate of Cr(VI) but lower removal rate of total Cr, from which it can be concluded that Cr(VI) was reduced to Cr(III) to a large extent and that Cr(III) then precipitated in S4 and S5 because of the alkaline environment.

#### Three-dimensional EKR experiments

**Figure 5:**
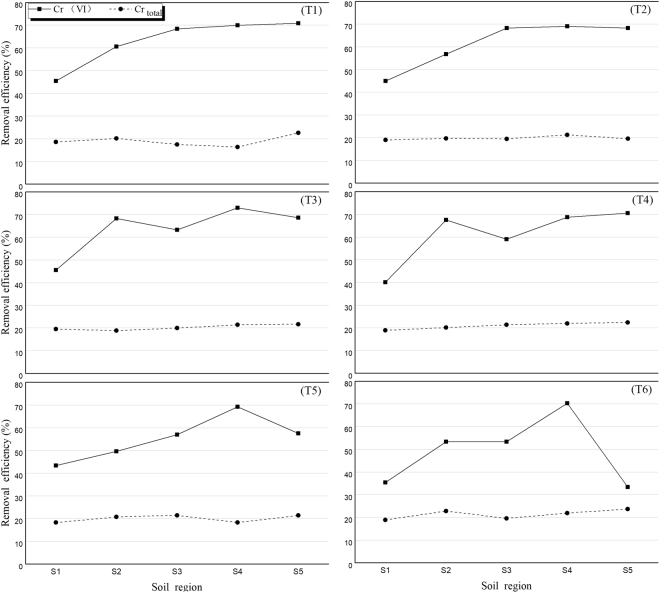
Effect of the dose ratio on the Cr removal efficiency in the 3D EKR experiment.

Figure 5 shows the Cr removal efficiency results from the 3D EKR experiments performed with various dose ratios. The removal efficiency of Cr(VI) after 5 d was significantly higher than that in the 2D EKR experiment. The results showed that the electrode particle ratio had varying effects on Cr(VI) removal efficiency but an insignificant effect on the total Cr. The highest Cr(VI) removal rate of 73.0% was observed for T3-S4. The detailed results and parameters of T1-T6 with the average removal rates are given in Table 4. The average removal rates defined as the average removal rate of Cr(VI) from the five sample regions (S1-S5) in a given test.

**Table 4:** Detailed results and parameters for Test 1–Test 6 in the 3D EKR treatment.

Test	Maximum removal			Minimum removal			Average removal rate
	Removal rate (%)	pH	Conductivity (mS/cm)	Removal rate (%)	pH	Conductivity (mS/cm)	
T1	70.9 (S5)	12.1	32.2	45.5 (S1)	4.0	56.3	63.0
T2	69.0 (S4)	11.8	30.8	44.9 (S1)	6.0	45.2	61.4
T3	73.0 (S4)	10.3	29.2	45.5 (S1)	3.6	55.9	63.7
T4	70.6 (S5)	11.2	37.0	40.0 (S1)	5.2	56.9	61.2
T5	69.2 (S4)	11.0	30.6	43.4 (S1)	5.0	48.3	55.3
T6	70.2 (S4)	11.9	35.2	33.3 (S5)	3.6	38.7	49.1

As shown in Table 4, the maximum removal rate of Cr(VI) was observed in regions S4 and S5. Meanwhile, the minimum removal ratio in S1 occurred at a low pH, which resulted in the adsorption of Cr(VI) and the oxidation of Cr(III) to Cr(VI). It was stated in previous reports that soils with an acidic environment exhibit high adsorption and slow migration of Cr(VI)^13,31^. Moreover, the higher accumulation and lower removal efficiency of Cr(VI) in S1 was due to its migration from the cathode to the anode. Region S1 had a higher electrical conductivity than regions S4 and S5, which was also due to the accumulation of free ions. In contrast to this result, regions S4 and S5 had lower ECs due to the migration of free ions from the cathode to the anode as well as the precipitation of reduced Cr in the presence of higher concentrations of OH^−^, which resulted from the electrolysis of water. The average removal rate among the different dosing ratios did not exhibit a regular trend, meaning that an increase in the dose ratio did not necessarily increase the removal rate i.e., T6, which was the highest dose ratio, had the lowest removal ratio among the six tests. A possible explanation for this phenomenon is discussed below. In the current study, the AC-Fe particles were uniformly distributed in the contaminated soil and functioned as a third electrode. These particles became polarized after a voltage was applied, having positive and negative poles and acting as an independent electrolytic cell^32^. Therefore, the introduction of too many electrode particles caused the formation of numerous small electric fields, which cluttered the electrolyser, thus hindering the electromigration of ions as a whole and thereby reducing the removal efficiency of Cr(VI). In addition, the excessive addition of electrode particles may result in a short-circuit current and increase the resistance during particle mass transfer, which would lead to a lower removal ratio of Cr(VI). So, based on the above discussion, the 5% particle dose was optimum in the 3D EKR tests.

### Effect of the remediation time on the removal efficiency

**Figure 6:**
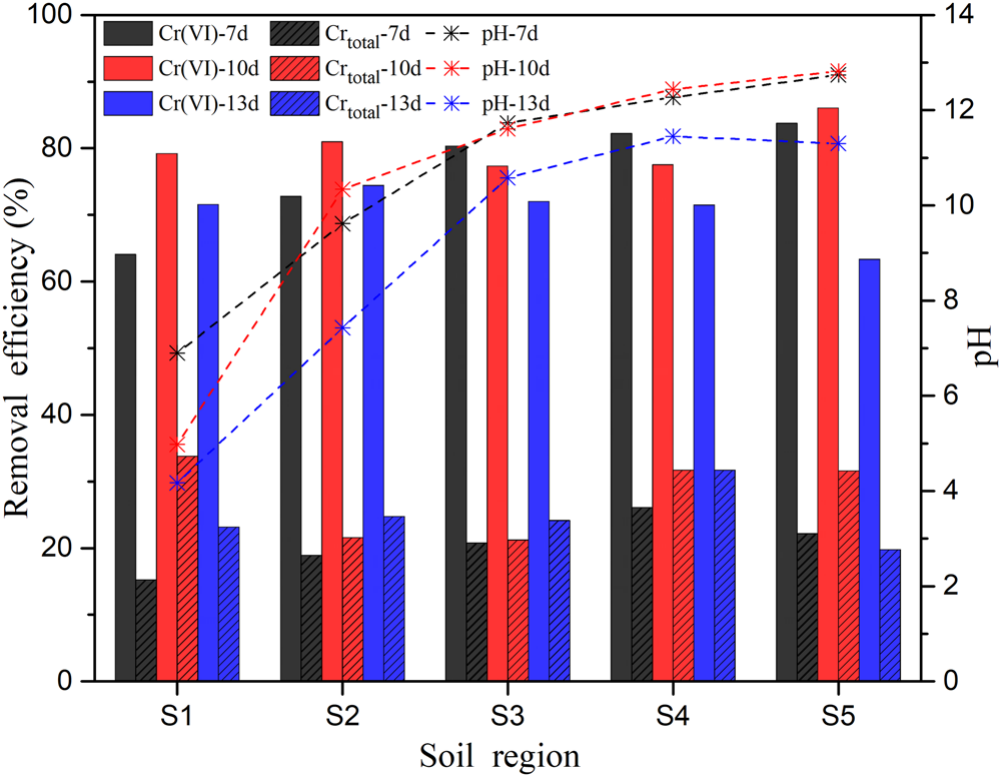
Effect of the remediation time on the Cr removal efficiency in the 3D EKR experiment.

Three-dimensional EKR tests were carried out to investigate the effect of the treatment time on the removal efficiency while using the optimum dose ratio (5%) based on Section 3.3. As shown in Fig. 6, the variations between the removal efficiencies of Cr(VI) and total Cr were significant, indicating that the reduction of Cr(VI) occurred. Previous studies have shown that an acidic environment is favourable for Cr(VI) reduction by Fe^0^ or Fe^2+^. In an acidic environment, Cr(VI) has a high redox potential and is easily reduced by electron donors like Fe^0^ or Fe^2+^, whereas under alkaline conditions, the formation of hydroxide is detrimental for Cr(VI) removal. In this study, a considerable amount of Cr(VI) was reduced in spite of the high pH, as illustrated in Fig. 6. Possible reasons for this result are discussed as below. The particle-based electrodes, as already discussed, behaved like independent electrolytic cells following polarization by the applied voltage in the 3D EKR experiments. These charged particles provided exchange sites for oppositely charged ions in a process called electrosorption^19,33^. Moreover, in their function as independent electrolytic cells, these particles generated H^+^ ions on one pole and thus formed a short-range acidic environment around this pole. One more reason for the high Cr(VI) removal under alkaline conditions was the adsorption of Cr by these particles due to their large surface area and porosity. So, these characteristics of electrosorption, short-range acidic environment and adsorption behaviour of the electrode particles (AC-Fe) created advantageous conditions for Cr(VI) reduction in the alkaline environment.

**Figure 7:**
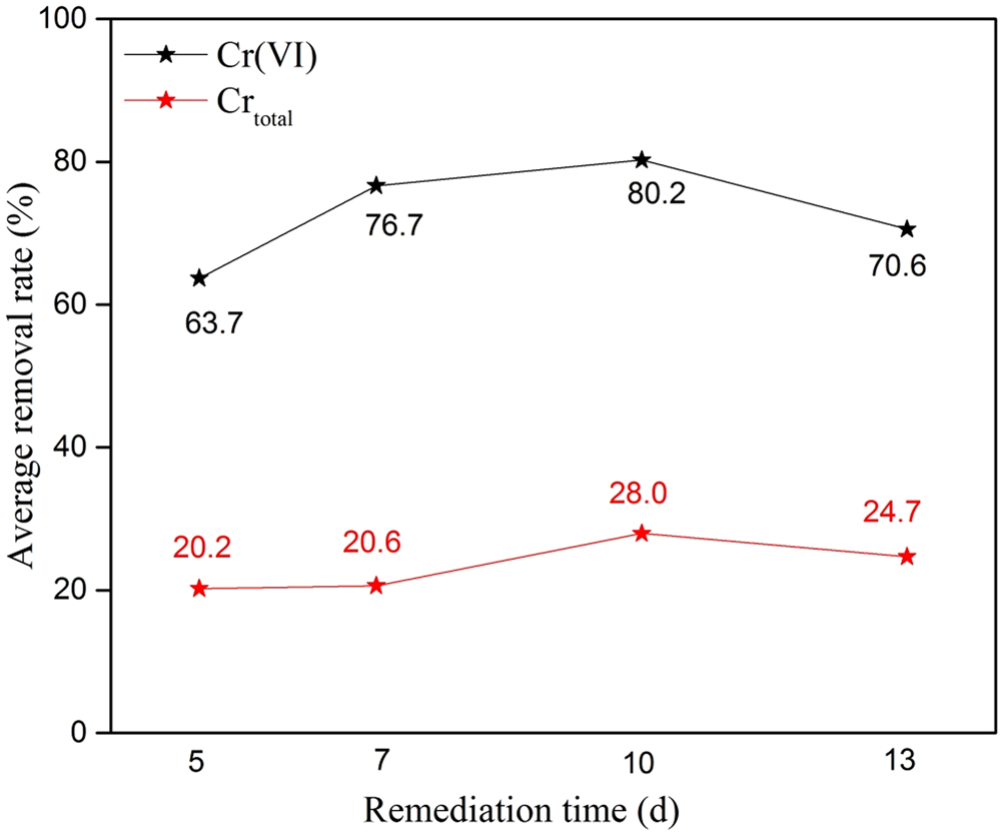
Average removal efficiency of Cr with respect to time.

Figure 7 shows the average removal ratio of Cr(VI) and total Cr with respect to time, and the same trend was observed for both Cr(VI) and total Cr. The average removal ratio of Cr increased with increasing remediation time up to a certain limit and then decreased. A remediation time of 10 d had the highest removal ratio (80.2%) compared to the ratios at 7 (76.7%) and 5 (63.7%) d. However, a decrease in the Cr removal ratio (70.6%) was observed after 13 d. A possible explanation for this phenomenon may be the adsorption equilibrium. Various ions were adsorbed by the particle electrodes in the electrolytic solution until the equilibrium state was reached. Therefore, in this study, the equilibrium state was attained at 10 d, and after that, the removal efficiency of Cr decreased. Another possible explanation may be the occurrence of a side reaction (Cr(III) oxidation by the generated hydroxyl radicals) after a certain amount of time, which affected the Cr(VI) removal ratio. Thus, it was concluded from Section 3.3 and 3.4 that a 5% dose ratio and a 10-d remediation time were the optimum conditions for Cr removal in this 3D EKR study.

### X-ray photoelectron spectroscopy analysis

**Figure 8:**
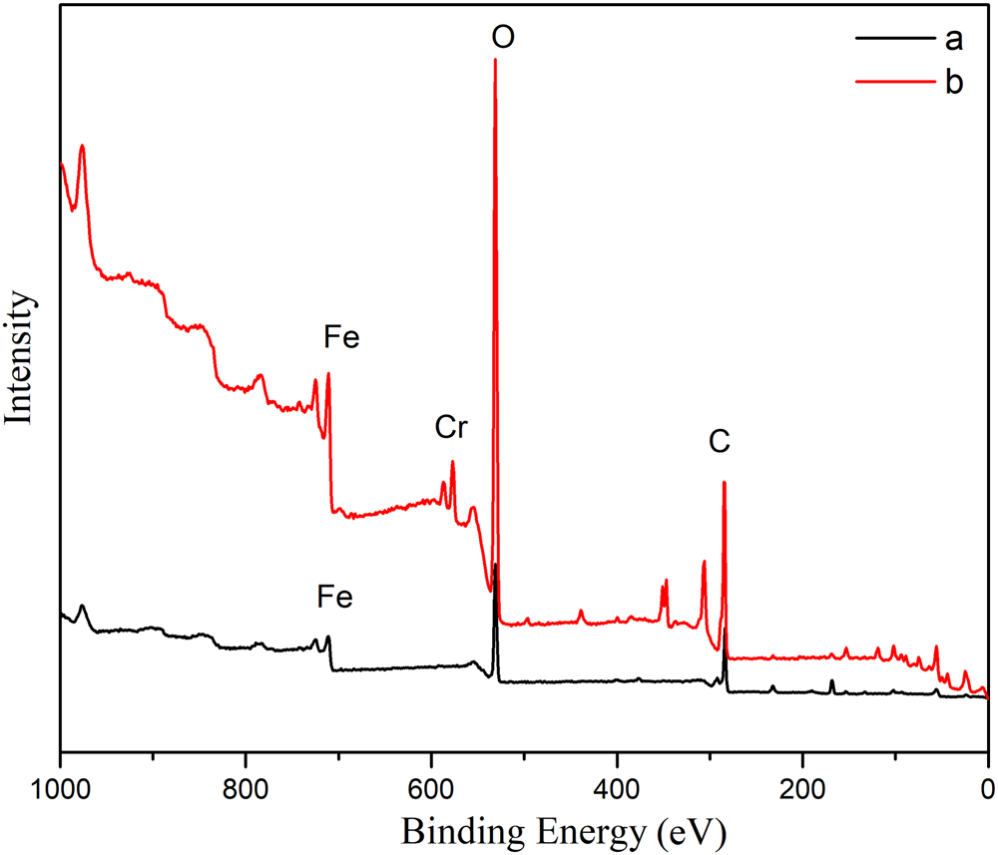
XPS data for the electrode particles (AC-Fe) (**a**) before and (**b**) after the experiment.

**Figure 9:**
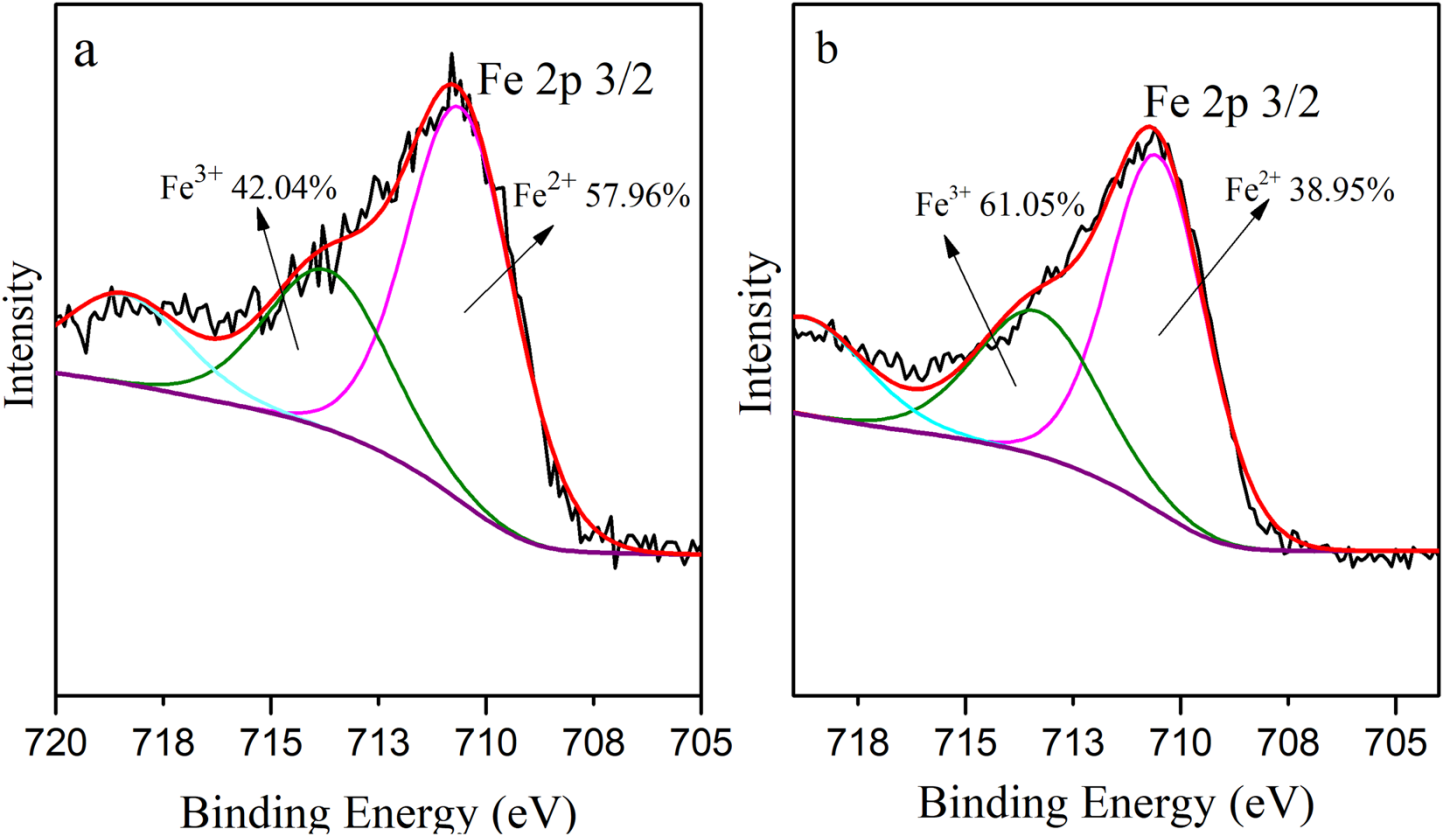
Fe 2p 3/2 XPS peak differentiation-imitating analysis of the electrode particles (AC-Fe) (**a**) before and (**b**) after the EKR experiment.

To examine the oxidation level of Fe^2+^ in the electrode particles (AC-Fe) and get a better understanding of the physicochemical reaction occurring during the EKR process, XPS analysis was performed on the electrode particles before and after the experiment. Furthermore, XPS was also performed to observe the valence state of Cr in the soil and on the electrode particles after remediation to verify its reduction during the EKR experiment. The spectra of the electrode particles (AC-Fe) before and after the experiment are shown in Fig. 8. The primary elements were observed on the basis of their corresponding binding energies, i.e., C at 531 eV (C 1 s) and O at 284 eV (O 1 s). A new peak for Cr was observed at a binding energy of 577 eV after the experiment, which verified the absorption of Cr on the electrode particles. To determine the valence state and relative content of Fe before and after the reaction, XPS peak differentiation-imitating analysis of the Fe 2p _3/2_ spectrum was conducted, as shown in Fig. 9(a) and (b). The photoelectron peaks observed at 710.8 eV and 710.6 eV corresponded to the binding energies of Fe 2p _3/2_, which suggested that the AC was covered by a layer of iron oxides/hydroxides of iron, such as FeO, Fe_3_O_4_ and Fe(OH)_3_^28,34^. Fe^3+^ was present on the AC was because of the inclusion of iron oxides during the synthesis of AC-Fe. XPS analysis indicated that the electrode particles were synthesized successfully and that part of the Fe^2+^ was oxidized to Fe^3+^. The percentages of Fe^3+^ and Fe^2+^ relative to the total Fe were calculated. By comparing Fig. 9(a) and (b), it can be seen that the relative content of Fe^2+^ after the reaction was reduced from 57.96% to 38.95%; meanwhile, that of Fe^3+^ increased from 42.04% to 61.05%, which implied that during the EKR experiment, the ferrous irons that were coupled with the activated carbon were likely to react with the Cr(VI) contaminants adsorbed on the surface of the electrode particles, causing a decrease in the Fe^2+^ content.

**Figure 10:**
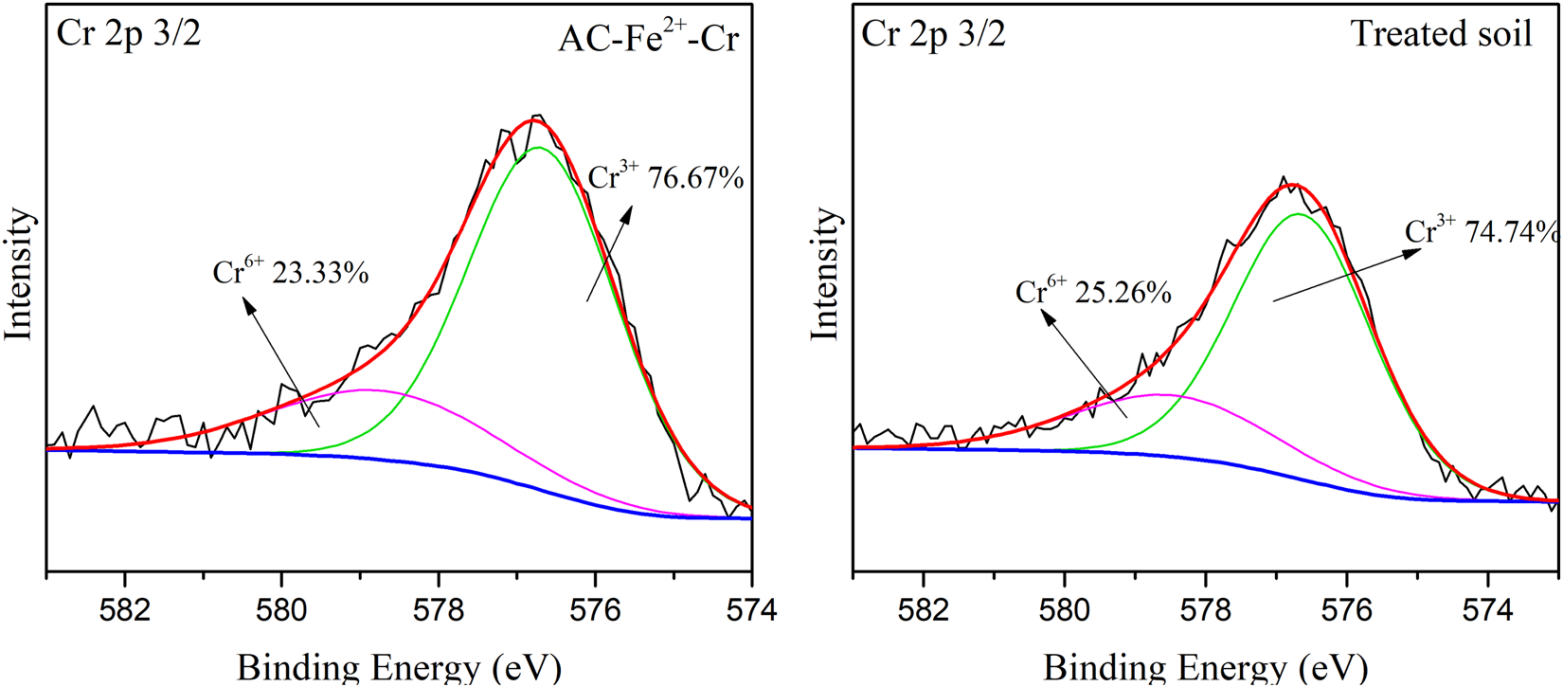
Cr 2p 3/2 spectra of the soil and electrode particles after remediation (AC-Fe-Cr).

To further investigate the removal mechanism of Cr(VI), the Cr 2p XPS spectra of the treated soil and the surface of the electrode particles were analysed, as shown in Fig. 10(a) and (b). The percentages of Cr(VI) and Cr(III) relative to the total Cr in the soil and on the electrode particles were calculated. The results indicated that the chromium present in the treated soil and adsorbed on the electrode particles predominantly existed in the trivalent form. However, the relative contents of Cr(VI) and Cr(III) in the treated soil were different from those adsorbed on the electrode particle surface. The electrode particles had 23.33% Cr(VI), which was slightly less than the Cr(VI) content of the treated soil (25.26%). This result verified that the reduction of Cr(VI) to Cr(III) occurred on the surface of the electrode particles.

### Removal mechanism of Cr in the 3D EKR experiments

Based on the above results, a possible mechanism for the removal of Cr(VI) in this 3D EKR system (using AC-Fe particles as the third electrode) was proposed. The removal process consisted of two strategies. One was common electromigration, in which Cr(VI) migrated from the cathode to the anode and was removed from the contaminated soil. The other process consisted of three stages: (i) Cr(VI) adsorption on the electrode particles due to the large specific surface area of AC-Fe and electrosorption; (ii) successful reduction of the adsorbed Cr(VI) to Cr(III) according to the reaction equations given below:

2 $${{\rm{Cr}}}^{6+}+{{\rm{3Fe}}}^{2+}+{\rm{AC}}\to {{\rm{Cr}}}^{3+}+{{\rm{3Fe}}}^{3+}+{\rm{AC}}$$

3 $${{\rm{Fe}}}^{2+}-{{\rm{e}}}^{-}\to {{\rm{Fe}}}^{3+}$$

4 $${\mathrm{Cr}}^{6+}+{{\rm{3e}}}^{-}+{\rm{AC}}\to {{\rm{Cr}}}^{3+}+{\rm{AC}}$$

and (iii) Cr(VI) adsorption on the electrode particles and subsequent reduction to Cr(III), where a portion of the Cr(III) species was simultaneously released into the soil because of the repulsion between same charges, the environmental pH, the migration to the cathode, and precipitation.

### Desorption and recycling of the three-dimensional electrode materials

**Figure 11:**
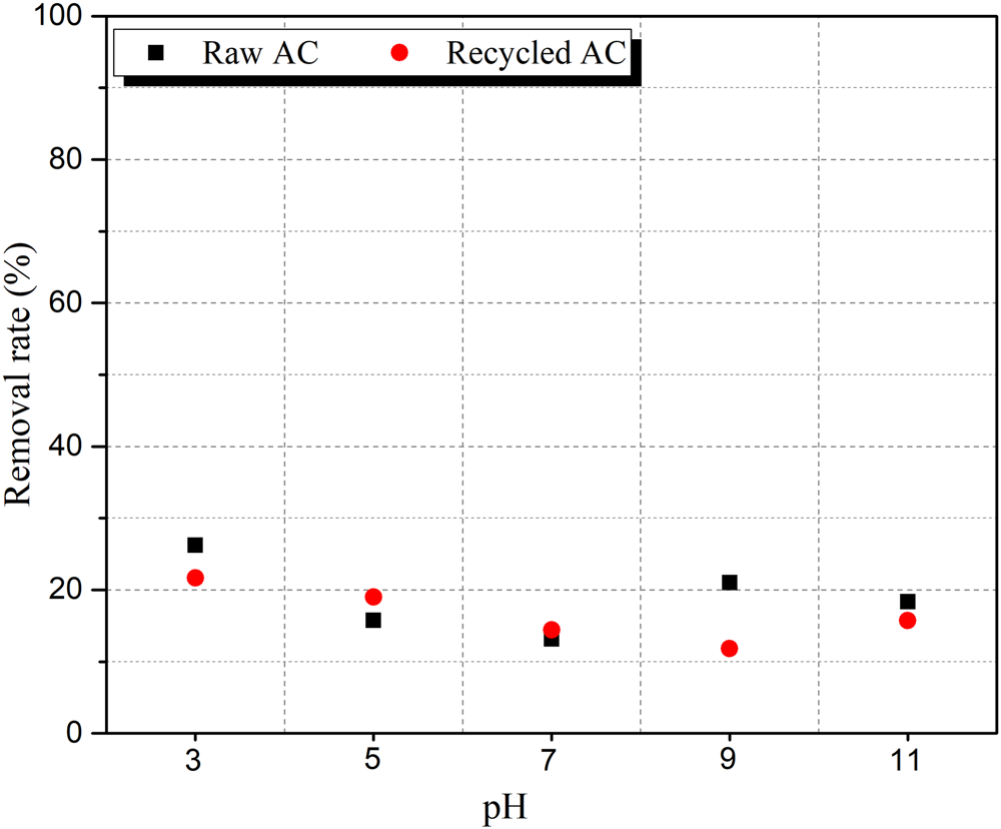
Aqueous equilibrium adsorption test results for raw and recycled AC particles.

After the experiment, the collected electrode particle were stirred in dilute hydrochloric acid for 5 h and dried (100 °C) after washing with deionized water until the pH of the filtrate pH was 7. The aqueous equilibrium adsorption test was performed according to the same procedures as those in Section 2.3 to examine the reusability of the electrode material. The result of this test is shown in Fig. 11. The adsorption capacity of the recycled AC decreased relative to that of raw AC due to the presence of contaminants in the pores. However, this decrease was very small. Therefore, the particles were capable of being reused in another EKR experiment process after recycling.

## Conclusions

In this study, the chromium removal efficiency of a 3D EKR system (using AC-Fe as the third electrode) was investigated. The results showed that 3D EKR more efficiently removed chromium than conventional EKR, and the removal efficiency depended upon the electrode particle dose ratio and the remediation time. The results of single-factor experiments showed that the highest Cr(VI) removal efficiency (80.2%) could be achieved by a 5% dose ratio and a 10-d remediation time in the presence of a 1 V/cm voltage gradient. The mechanisms involved in Cr(VI) removal were electromigration of Cr(VI) and Cr(III), adsorption/electrosorption of Cr(VI) on the electrode particles and reduction of Cr(VI) to Cr(III).
